# The first study on the seroprevalence of *Anaplasma* spp. in small ruminants and assessment of associated risk factors in North Egypt

**DOI:** 10.14202/vetworld.2022.1221-1227

**Published:** 2022-05-20

**Authors:** Abdelfattah Selim, Kotb A. Attia, Roua A. Alsubki, Fatima Albohairy, Itoh Kimiko, Mourad Ben Said

**Affiliations:** 1 Department of Animal Medicine (Infectious Diseases), Faculty of Veterinary Medicine, Benha University, Toukh 13736, Egypt; 2 Department of Biochemistry, College of Science, King Saud University, P.O. Box 2455, Riyadh 11451, Riyadh, Saudi Arabia; 3 Department of Clinical Laboratory Science, College of Applied Medical Sciences, King Saud University, P.O. Box 2455, Riyadh 11451, Riyadh, Saudi Arabia; 4 Department of Extramural Research, Health Sciences Research Center, Princess Nourah bint Abdulrahman University, P.O. Box 84428, Riyadh 11671, Saudi Arabia; 5 Institute of Science and Technology, Niigata University, Ikarashi-2, Nishi-ku, Niigata 950-2181, Japan; 6 Department of Basic Sciences, Higher Institute of Biotechnology of Sidi Thabet, University of Manouba, Manouba 2010, Tunisia; 7 Laboratory of Microbiology at the National School of Veterinary Medicine of Sidi Thabet, University of Manouba, Manouba 2010, Tunisia

**Keywords:** *Anaplasma* spp, competitive enzyme-linked immunosorbent assay, Egypt, risk factors, small ruminants

## Abstract

**Background and Aim:**

Ovine anaplasmosis is a rickettsial disease caused by *Anaplasma* spp. These Gram-negative intracellular bacteria are mainly transmitted by ticks and infected blood cells of caprine, ovine, and wild small ruminants. At present, epidemiological data on anaplasmosis in cattle, dogs, and camels in Egypt are available, but the data about *Anaplasma* spp. in sheep and goat are scarce. This study aimed to determine the seroprevalence of *Anaplasma* spp. in small ruminants and assess the associated risk factors.

**Materials and Methods:**

A cross-sectional study was performed to investigate the seroprevalence of *Anaplasma* spp. in 300 sheep and 300 goats from four governorates in North Egypt using a commercial competitive enzyme-linked immunosorbent assays kit, and the associated risk factors for the infection were evaluated.

**Results:**

Overall, the seroprevalence of anti-*Anaplasma* antibodies was 18.3% and 21.3% in sheep and goats, respectively. A multivariable logistic regression model was used to determine the association between risk factors and *Anaplasma* spp. infection.

**Conclusion:**

Age, animal husbandry, acaricide use, tick infestation, and contact with cattle were the primary risk factors for *Anaplasma* seropositivity. This study confirms the presence of antibodies against *Anaplasma* spp. in small ruminants from Egypt. This is the first study to assess the associated risk factors for *Anaplasma* infection in small ruminants from Egypt. Further studies are needed to improve the understanding of the associated disease factors, facilitating the development of new procedures for control of anaplasmosis in livestock.

## Introduction

*Anaplasma* spp. is an obligate intracellular bacterium from the Rickettsiales order and *Anaplasmataceae* family. Anaplasmosis is a tick-borne disease that affects various animal species, including small ruminants, and the bacteria can infect red and white blood cells [1-3]. Anaplasmosis is an endemic disease that causes hemolytic anemia in various tropical and subtropical regions worldwide [4-6]. Animals of all ages, including goats and sheep, are susceptible to *Anaplasma ovis* infection. However, goats are more susceptible to infection than sheep. Clinical signs common in goats and sheep generally serve as a subclinical reservoir in their herds [7,8].

Tick vectors are the primary mode of transmission for the bacterium, whereas *Rhipicephalus*, *Ixodes*, *Amblyomma*, and *Dermacentor* ticks are the most common ticks that transmit *Anaplasma* spp. [9-12]. Transmission by mechanical routes from contaminated needles or surgical instruments used in unsanitary conditions or, by biting flies is also possible.

Acute anaplasmosis manifested by fever, anemia, depression, decreased body weight, reduction in milk production, abortion, dyspnea, and deterioration in the physical condition can lead to death [13]. Immunocompromised animals, either to splenectomy or concomitant microbial infection, are more susceptible to clinical anaplasmosis, and infected animals are long-term reservoirs [14]. Furthermore, the severity of an *Anaplasma* infection is also influenced by other spatio-temporal factors, such as bacterial load, vector habitat, bacterial populations, grazing system, management, and hygienic practices [15].

Direct microscopic blood smears are commonly used to identify *A. ovis*-infected sheep during the acute phase of anaplasmosis. Despite its limited sensitivity, light microscopy is the gold standard but it requires an expert examiner and is time-consuming [16]. In addition, the timing of blood sampling for microscopic examination is crucial since this test must be performed when clinical signs appear during the early acute stage of the disease before the administration of drugs [17]. Since antibodies can be detected at all phases of anaplasmosis infection in animals, serological techniques are advantageous over microscopic investigation in many cases [18]. Serological approaches may be limited in carrier animals due to their lack of specificity, sensitivity, reproducibility, and interpretation [19-24]. Since competitive enzyme-linked immunosorbent assays (cELISAs) have high sensitivity and specificity, it offers more advantages than other serological tests such as the agglutination test, complement fixation test, and immunofluorescent assay [25-27].

In Egypt, few studies have been reported for *Anaplasma* spp. in cattle, buffalo, and camels [2,3]. At present, no research has investigated *Anaplasma* spp. in small ruminants.

Therefore, this study aimed to estimate the seroprevalence of *Anaplasma* spp. in small ruminants located in four governorates of North Egypt. It evaluates the risk factors that could be implicated in *Anaplasma* spp. infection.

## Materials and Methods

### Ethical approval and Informed consent

All procedures involving the handling and collection of blood samples were approved by the ethical committee for Animal Experiment of Benha University (Approval No: BUFVTM) and informed consent was obtained from owners.

### Study period and location

**Figure-1:**
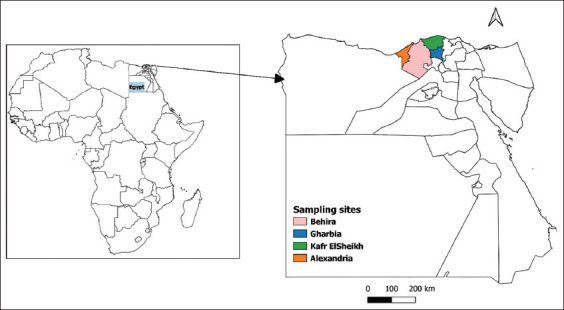
Map depicts governorates where the study animals were sampled [Source: Map generated by QGIS software].

The study was conducted from January to December 2020 in four governorates (Alexandria, Behira, Kafr El Sheikh, and Gharbia) located in North Egypt (Figure-1). The climate of the selected areas is characterized by a hot Mediterranean climate in the summer. This season is usually warm with an average temperature of 25°C, while the winter is cold, windy with an average temperature of 15°C, and an average rainfall around 200 mm.

### Samples collection

The sample size required for the present study was determined using Thrusfield formula [28]. A total of 600 blood samples (300 sheep and 300 goats) were collected randomly and represented the four regions in the study. A volume of 5 mL of blood was drained from the jugular vein of each animal using sterile tubes without anticoagulant. Sera were separated from each blood sample by centrifugation at 3500× *g* for 10 min and kept at –20°C for serological analysis.

The age, gender, and breeding system were recorded for each animal. In addition, data on acaricide use, tick infestation, and contact with cattle were collected to evaluate their risk of infection.

### Serological analysis

Antibodies against *Anaplasma* spp. were identified using a commercial cELISA kit (*Anaplasma* antibody test kit, cELISA; VMRD Inc., Pullman, WA, USA), according to the manufacturer’s instructions. This cELISA was approved for the detection of antibodies directed against the *MSP5* protein of *Anaplasma* *centrale*, *Anaplasma marginale*, and *A. ovis* [29]. The optical density of the plate was read by a microplate reader at 620 nm. Results were calculated as [1−(sample OD620/OD620 of negative control)] 100 and reported as the percent inhibition (% I). The sample was considered positive if the % I was <30%.

### Statistical analysis

Data were analyzed with Statistical Package for the Social Sciences software ver. 24.0 (IBM Corp., NY, USA). Chi-square test was performed to compare seropositivity to *Anaplasma* spp., p≤0.05 was considered statistically significant. Univariable analysis was used to determine the relationship between seropositivity of *Anaplasma* spp. in sheep and goats and variables of the geographic regions (Alexandria, Behira, Kafr El Sheikh, and Gharbia), age (<2 years and ≥2 years), gender (male and female), animal breeding (stable, nomadic, and nomadic and pasture), acaricide use (regular and irregular), tick infestation, and contact with cattle. Variables with p<0.2 were examined using a multivariable logistic regression model to identify risk factors, odds ratios, and confidence intervals for each significant variable.

## Results

*Anaplasma* seropositivity was found in 18.3% (55/300) of sheep and 21.3% (64/300) of goats examined in four governorates belonging to North Egypt. The results revealed that locality (p=0.92 and 0.41, respectively) and the gender of sheep and goats (p=0.50 and 0.37, respectively) had no significant association with *Anaplasma* exposure risk.

The highest seroprevalence rate of *Anaplasma* spp. was observed in Alexandria for sheep and in Gharbia for goats (Table-1). Furthermore, the seropositivity of *Anaplasma* spp. increased in female sheep (19.5%) and male goats (24.7%), but the difference in seroprevalence rates between genders was not statistically significant for the two small ruminant species (Table-1).

**Table 1:** Univariable analysis for risk factors associated with *Anaplasma* spp. seroprevalence in sheep and goats.

Variable	Sheep					Goats				
		
Locality	Number of samples	Number of positive	%	95%CI	Statistic	Number of samples	Number of positive	%	95%CI	Statistic
Alexandria	80	16	20.0	12.7-30.1	χ^2^=0.481 df=3 p=0.923	82	19	23.2	15.4-33.4	χ^2^=2.903 df=3 p=0.407
Behira	70	11	15.7	9-25.9		65	9	13.8	7.5-24.3	
Kafr El Sheikh	80	15	18.8	11.7-28.6		93	21	22.6	15.3-32.1	
Gharbia	70	13	18.6	11.2-29.2		60	15	25.0	15.7-37.2	
Age										
<2 years	100	8	8.0	4.1-15	χ^2^=10.698 df=1 p=0.001*	90	10	11.1	6.2-19.3	χ^2^=8.005 df=1 p=0.005*
>2 years	200	47	23.5	18.2-29.8		210	54	25.7	20.3-32.1	
Gender										
Male	110	18	16.4	11.7-26.7	χ^2^=0.450 df=1 p=0.50	85	21	24.7	16.7-34.8	χ^2^=0.804 df=1 p=0.37
Female	190	37	19.5	14.5-25.7		215	43	20.0	15.2-25.8	
Animal breeding										
Stable	40	7	17.5	8.7-31.9	χ^2^=6.385 df=2 p=0.04*	55	7	12.7	6.3-24.1	χ^2^=5.093 df=2 p=0.03*
Nomadic	120	30	25.0	18.1-33.4		180	46	25.6	19.7-32.4	
Nomadic and pasture	140	18	12.9	8.3-19.4		65	11	16.9	9.7-27.8	
Acaricides use										
Regular	50	3	6.0	2.1-16.2	χ^2^=6.096 df=1 p=0.014*	55	6	10.9	5.1-21.8	χ^2^=4.361 df=1 p=0.037*
Irregular	250	52	20.8	16.2-26.3		245	58	23.7	18.8-29.4	
Tick infestation										
Infested	130	33	25.4	18.7-33.5	χ^2^=7.618 df=1 p=0.006*	105	46	43.8	34.7-53.4	χ^2^=48.626 df=1 p≥0.0001*
Non-infested	170	22	12.9	8.7-18.8		195	18	9.2	5.9-14.1	
Contact with cattle										
Yes	195	42	21.5	16.4-27.8	χ^2^=4.361 df=1 p=0.037*	175	50	28.6	22.4-35.7	χ^2^=13.111 df=1 p≥0.0001*
No	105	13	12.4	7.4-20.1		125	14	11.2	6.8-17.9	

* The result is significant at p<0.05. CI=Confidence interval

In this study, age, animal breeding, acaricide use, tick infestation, and contact with cattle have a significant role in *Anaplasma* spp. seropositivity in sheep and goats (Table-1). Adult sheep and goats were more likely to be *Anaplasma* seropositive (23.5% and 25.7%, respectively) than younger ages (8% and 11.1%, respectively) (p=0.5 and 0.37, respectively).

The probability of infection was affected by the animal breeding system, particularly the nomadic one, while 25% of sheep and 25.6% of goats were seropositive for *Anaplasma* spp. in a nomadic husbandry system (p=0.04 and 0.03, respectively).

The seroprevalence of *Anaplasma* spp. in sheep and goats significantly increased with irregular use of acaricides (20.8% and 23.7%), infestation by ticks (25.4% and 43.8%), and in contact with cattle (21.5% and 28.6%), respectively (Table-1). The multivariable binary logistic regression for all variables had p<0.2 for sheep and goats (Table-2).

**Table 2:** Multivariable analysis for risk factors associated with seroprevalence of anaplasmosis in sheep and goats.

Variable	B	SE	OR	95% CI for OR		p-value

				Lower	Upper	
Sheep						
Age						
≥2 years	1.076	0.418	2.93	1.29	6.66	0.010
Breeding						
Stable	0.527	0.511	1.69	0.62	4.61	0.302
Nomadic	0.687	0.346	1.99	1.01	3.92	0.047
Acaricides use						
Irregular	1.123	0.564	3.07	1.02	9.29	0.047
Tick infestation						
Infested	0.813	0.318	2.26	1.21	4.20	0.010
Contact with cattle						
Yes	0.575	0.359	1.78	0.88	3.59	0.110
Goats						
Age						
≥2 years	1.025	0.406	2.79	1.26	6.18	0.012
Breeding						
Nomadic	0.521	0.477	1.68	0.66	4.29	0.275
Nomadic and pasture	−0.056	0.580	0.95	0.30	2.95	0.924
Acaricides use						
Irregular	1.035	0.504	2.82	1.05	7.56	0.040
Tick infestation						
Infested	1.957	0.334	7.08	3.68	13.63	>0.0001
Contact with cattle						
Yes	1.001	0.364	2.72	1.33	5.55	0.006

B=Wald statistic, SE=Standard error, CI=Confidence interval, OR=Odds ratio

The probability of antibodies against *Anaplasma* spp. was 2.93-fold in older sheep (≥2 years old), 1.99-fold among animals kept in a nomadic system, 3.07-fold with irregular acaricide use, 2.26-fold in animals infested with ticks, and 1.78-fold in small ruminants contacted with cattle versus others (Table-2). Moreover, adult goats (≥2 years old) and goats kept in a nomadic system were 2.79- and 1.68-fold more likely to have *Anaplasma* spp. antibodies than younger animals and goats kept in stables or nomadic and pasture husbandry (Table-2).

## Discussion

Anaplasmosis is a tick-borne disease that affects various domestic ruminants, including sheep and goats, and is widely distributed in tropical and subtropical regions [2-4]. In Egypt, despite the detection of *Anaplasma* spp. infection in dogs [30], cattle [31], and camels [32], there are no studies about these bacteria in sheep and goats. This is the first survey on *Anaplasma* infection in small ruminants from Egypt.

This study investigated anti-*Anaplasma* antibodies in 600 blood samples collected from 300 sheep and 300 goats using a cELISA assay based on MSP5 antigen. This tool has more advantages over other serological tests, with a sensitivity of 96% and a specificity of 95% for the detection of *Anaplasma* spp. antibodies.

Based on the current results, the seroprevalence of *Anaplasma* spp. in sheep and goats was 18.3% and 21.3%, respectively, in four governorates of North Egypt. The seroprevalence showed an insignificant disparity between different studied areas. Compared to the previous study conducted by Khezri [19], the seroprevalence in sheep in the present study remains higher than what was reported in Iran 6.4%, but it was similar to the study conducted in Pakistan with a seroprevalence rate of 19% [8].

For goats, the seroprevalence rate is consistent with the previous seroprevalence rate observed in Pakistan (25%) [8], while it was lower than the seroprevalence rate estimated in Botswana (88%) [33] and Jordan (82%) [34]. However, the difference between seroprevalence rates in these countries could be due to variations of several factors such as the sampling process, the number of examined animals, the bioclimate, and the kind of tests used [27,31].

Based on the previous literature, direct microscopic smear and PCR techniques were able to detect *Anaplasma* infection in sheep and goats, particularly during the acute phase of infection, but there was great variation between results of these two methods. However, PCR is more sensitive and accurate than microscopy [35,36]. Interestingly, the variation between the previous studies based on the cELISA technique is very low when compared to other methods. However, a cELISA could detect antibodies after exposure to infection, but other methods could be used to investigate the acute infection [37].

This study showed that seropositivity to *Anaplasma* spp. was more common in older sheep and goats than in younger ones, which corresponds to the previous ‘study conducted by Khan *et al*. [8]. These results could be explained by the fact that older animals are more likely to be infested by arthropods due to their longer lifespans and exposure to more vector seasons.

According to the present findings, animal gender did not significantly affect *Anaplasma* infection, and females were more likely to acquire the infection than males. Similar findings were previously reported by Khan *et al*. [8] and Rajput *et al*. [38]. This phenomenon may have contributed to certain stress factors associated with females, such as pregnancy, parturition, concurrent infections like parasitism, or malnutrition [30,31].

The highest seroprevalence rate of *Anaplasma* spp. was observed among animals raised in nomadic breeding [32]. This observation could be due to disease in carrier animals that showed no clinical signs but plays a role in the spread of infection among susceptible animals [39]. In addition, animals kept in a stable receive more veterinary care and are kept in a clean environment most of the time compared to animals kept in an open pasture. Compared to older studies, the prevalence of anaplasmosis in sheep and goats significantly increased in tick-infested animals and associated with the lack of acaricide use [6,8]. This explains the fact that the transmission of *Anaplasma* spp. mainly occurred by ticks and mechanically by bites from flies [40].

Further, sheep and goats kept in contact with cattle showed a higher risk of being infected with *Anaplasma* spp. than animals kept separately [32]. This study had some limitations since the blood samples were collected from four governorates and did not represent a large study area in Egypt, and the samples were collected from animals living under different management conditions that affect the distribution of the disease.

## Conclusion

Anaplasmosis spreads in cattle and dogs and the results of this study confirm the occurrence of the disease in small ruminants. Specific and efficient diagnostic techniques are needed to investigate early infection and identify carrier animals to reduce economic losses. Furthermore, the search for risk factors associated with Anaplasma infections and the awareness of farmers and decision-makers will help establish an effective control program for the disease.

## Authors’ Contributions

AS, KA, RA, FA, and MBS: Conceptualization, methodology, formal analysis, investigation, resources, data curation, writing-original draft preparation. AS and MBS: Writing-review and editing. AS, KA, RA, and FA: Project administration. AS, KA, RA, and FA: Funding acquisition. All authors have read and approved the final manuscript.
